# Adoption of Sustainable Agriculture Practices in Banana Farm Production: A Study from the Sindh Region of Pakistan

**DOI:** 10.3390/ijerph17103714

**Published:** 2020-05-25

**Authors:** Rafay Waseem, Gershom Endelani Mwalupaso, Faria Waseem, Humayoon Khan, Ghulam Mustafa Panhwar, Yangyan Shi

**Affiliations:** 1 College of Economics and Management, Nanjing Agricultural University, Nanjing 210095, China; rafaysami16@outlook.com (R.W.); rinscod@gmail.com (G.E.M.); fariawaseem.sau@gmail.com (F.W.); khanhumayoon88@yahoo.com (H.K.); mghulam83@gmail.com (G.M.P.); 2 Department of Agriculture and Agribusiness, Prince G Academy and Consultancy, Kabwe 10101, Zambia; 3 China Centre for Food Security Studies, Nanjing Agricultural University, Nanjing 210095, China; 4 Department of Management, Macquarie Business School, Macquarie University, Sydney 2121, Australia

**Keywords:** socioeconomic factors, psychosocial factors, theory of planned behavior, banana production, sustainable agricultural practices, Pakistan

## Abstract

The aim of this study was to highlight the importance of socioeconomic and psychosocial factors in the adoption of sustainable agricultural practices (SAPs) in banana farm production. To this end, data from 300 randomly selected farm households from Pakistan were collected through a structured self-report questionnaire. Using logistic regression (LR) and structural equation modeling (SEM), socioeconomic and psychosocial effects were evaluated. The results show that economic status, watching agricultural training programs, newspaper and radio awareness campaigns, participation in extension programs, perceptions of sustainable agriculture and the feasibility of SAPs were significant factors in farmers’ adoption of sustainable agriculture practices. Also, consistent with the theory of planned behavior (TPB), all its dimensions (attitude, subjective norms and perceived behavioral control) affected the adoption of SAPs. This finding highlights the importance of socioeconomic and psychosocial factors in promoting sustainable agricultural practice among banana production farmers. This is the first study which attempts to provide empirical evidence using a robust procedure (two models—LR and SEM). The practical implication is that, when socioeconomic and psychosocial factors are well supported by satisfactory policy measures, SAP adoption is more than likely, which eventually increases farmers’ adaptive capacity to the changing environment. Ultimately, this leads to sustainable banana production, which has great potential to contribute towards poverty eradication.

## 1. Introduction

Globally, smallholder farming systems significantly contribute to poverty alleviation and rural development [1]. Particularly in developing countries like Pakistan, national development plans stress the importance of investments in smallholder farming in order to ensure food security and reduce poverty in developing economies. However, the negative impact of modern agriculture on human health, the natural environment and resources have led to the growth and spread of a feature called sustainable agriculture [2]—a type of agriculture that focuses on the long-term production of crops and livestock while having minimal effects on the environment, as described by Mwalupaso [3]. It facilitates better use of natural resources, and is helpful in protecting the environment and reducing the use of external inputs [4]. The adoption of sustainable agricultural practices (SAPs) allows for farm workers to explore and accumulate more knowledge and skills on how best to achieve sustainable agriculture.

To a very large extent, SAPs are subjectively assessed for their compatibility with the existing values, past experiences, and needs of potential adopters. Fitting more of these criteria is likely to result in adoption [5]. However, some scholars hold the view that SAPs could be difficult to understand and/or use as greater complexity is likely to eschew their adoption [6,7]. According to Tatlıdil, Boz [8] adoption is a decision to make full use of an innovation as the best course of action available. In respect to SAPs, the decision-making involves multi-dimensional considerations [9]. They can be grouped into (i) socioeconomic factors, (ii) agro-ecological factors, (iii) institutional factors, (iv) informational factors, (v) perceived attributes and (vi) psychosocial factors. This study zeros in on socioeconomic factors (social and economic conditions relevant to the farm decision-maker [10] which represent human capital, farmers’ capacity and ability) and psychosocial factors (psychological attitude formations towards a behavior). Regarding the latter, widely studied factors include attitude and intention [11,12]. A positive attitude represents a favorable response towards an object, while intention indicates that farmers are willing to perform a behavior. Therefore, expression of intentionality is likely to see farmers realize the behavior.

Arguably, the importance of sustainable agriculture varies from farmer to farmer, and it is affected by their economic assets and behavior. Thus, modeling farmers’ opinions about practical agriculture and anticipating the determinants is pivotal for planning expansion programs to attain feasible, sustainable agricultural advancement [13]. Various scholars have found that the adoption of SAPs had a significant impact on farmers’ productivity [14,15]. However, very limited empirical research has focused on the farmers’ behavioral intentions to adopt SAPs [16]. A thorough review of previous literature reveals that different scholars investigated SAP adoption in different countries. However, until now there is hardly a study on this cardinal topic under the domain of the Pakistani agriculture sector. More importantly, existing studies also reveal that efforts to understand attitude, subjective norms (SN) and perceived behavior control (PBC) were neglected in studying the adoption of SAPs [17,18,19,20]. Table 1 presents the details. Therefore, it is important to understand whether socioeconomic and psychosocial factors impact the adoption of SAPs for crucial crops that are significant in augmenting farmers’ income and improving household food security status. SAPs have great potential to simultaneously confront cropland degradation, low agricultural productivity and poverty because they play a crucial role in the maintenance of resilient agroecosystems [3,21].

For example, in Pakistan, bananas are one of the largest horticultural crops [27]. Most bananas are consumed locally, and only a small fraction of the fruit is transported to Afghanistan, Azerbaijan and Iran. Farmers consider the cultivation of bananas an appealing option because they are cultivated throughout the year, and also because of the existence of enormous global markets [28,29,30]. Also, the banana is a heavyweight when it comes to nutrition. It is loaded with essential vitamins and minerals such as potassium, calcium, manganese, magnesium, iron, folate, niacin, riboflavin and B6. These all contribute to the proper functioning of the body and keeping you healthy. Thus, their production contributes to human nutrition. The banana is a major fruit crop of Pakistan. It is grown on 34,800 hectares, with a production of 154,800 tons. It is mainly grown in the Sindh province, where the soil and climatic conditions are favorable for its successful cultivation. The total share of the Sindh province alone in its cultivation is 87%. However, over the past few years, banana growers, exporters, suppliers and public leaders have raised concern over the sustainability of production, due to the agricultural practices adopted by many farmers—the overuse of chemicals to control disease and pests [31,32]. The major debate has been how to avoid production waste and realize an increase in high-quality bananas. While SAPs are deemed a solution, the adoption levels remain very low. One plausible explanation could be that socioeconomic and psychosocial factors directly associated with SAP adoption are poorly understood. Therefore, this study aims to address the role of these factors in stimulating SAP adoption in banana production by employing a robust analytical framework that cogently informs policy.

Consequently, the study makes a contribution to literature in the following way. The banana industry in many countries has a perceived image of overusing pesticides and fertilizers, due partly to the clonal nature of cultivars used for commercial production. The issue about how to get particularly resource poor, smallholder growers to change practices is an ongoing challenge, and is worthy of investigation. While there is a need for farmers to improve practices worldwide, leading to improved financial security and the protection of valuable environmental resources, empirical evidence is limited. To the best of our knowledge, this is the first study addressing this important subject.

The remainder of this paper is structured as follows: Section 2 provides the materials and methods which contain the description of the data, conceptual framework and empirical strategy; Section 3 presents the results, while the discussion is provided in Section 4; finally, the conclusion is provided in Section 5.

## 2. Materials and Methods

### 2.1. Description of Study Area and Data

**Figure 1:**
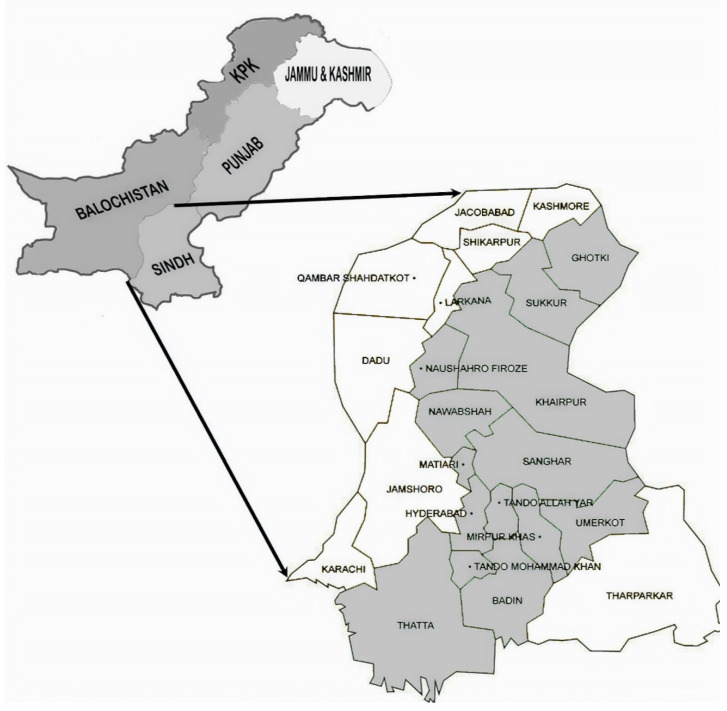
Study areas of Sindh, Pakistan.

The present study was conducted in the Sindh province of Pakistan (Figure 1), which is one of the major banana producing areas of the country. The soils and climatic conditions are favorable for successful banana cultivation, which facilities a significant contribution to the national food basket. Thatta, Hyderabad, Badin, Mirpurkhas, Tando Allahyar, Matiari, Tando Muhammad Khan, Sangar, Naushero Feroz and Nawabshah are the major districts in Sindh where bananas are grown. The province also grows other crops, and is one of the major producers of rice and wheat.

The data used in this study was collected through a household survey conducted from January to July 2019. A two stage sampling procedure was employed to select the sample. First, the province was selected on the basis of being a banana growing region. Finally, 300 banana farmers were randomly chosen using the farmers’ list of banana growers from the Ministry of Agriculture in the study area. Like most farming communities in Pakistan, female participation is generally low, due to cultural barriers for females in agriculture participation, as indicated by Rasheed [33]. The questionnaire was the primary source of data collection. To ensure quality data collection, the questionnaire was structured and pretested, and experienced and trained enumerators were employed. The SAPs adopted in the area can be broken down into the following categories: land management, fertiliser practices, pesticide practices, crop management, harvest management, post-harvest and marketing. Table 2 details the adoption rates.

### 2.2. Conceptual Framework and Hypothesis Development

**Figure 2:**
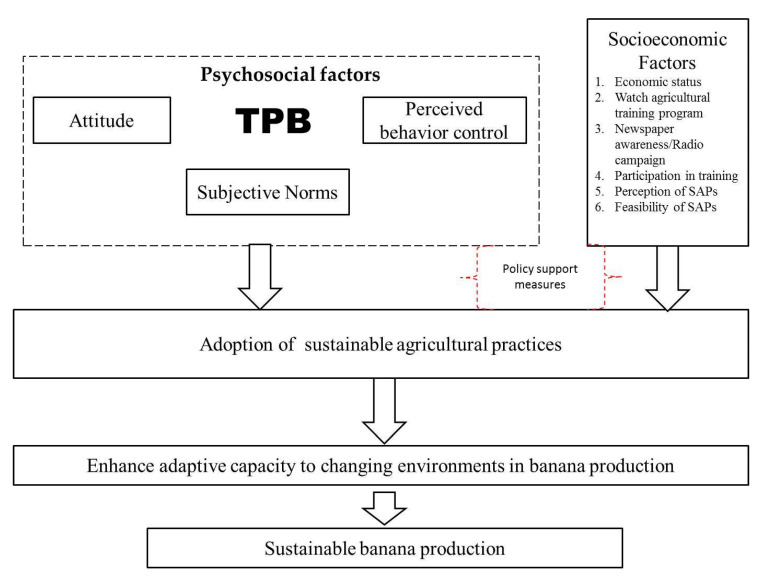
Conceptual framework. Notes: TPB and SAPs stand for Theory of Planned Behavior and Sustainable Agricultural Practices.

The conceptual framework of the present study is based on the theory of planned behavior (TPB). As put forward by Ajzen [34], TPB explains the role of attitudes, subjective norms (SN) and perceived behavioral control (PBC) in influencing observed behavior. SN implies social pressure to adhere to a certain conduct, while PBC denotes the extent to which the individual perceives that they have control over engaging in the behavior. In this study, behavior is the adoption of sustainable agricultural practices. The aforementioned constitute psychosocial factors that may influence the adoption of SAPs, as depicted in the Figure 2.

Regarding socioeconomic factors, we postulate that socioeconomic status, watching agricultural training programs, newspaper and radio awareness campaigns, participation in sustainable agricultural training extension courses, sustainable agricultural perceptions and the feasibility of sustainable agricultural practices are major drivers of adoption in Pakistan. However, for both factors to be effective, policy support measures must be in place. Eventually, this would help banana farmers to enhance their capacity to adapt to the changing environment and ensure production is productive.

Prior study regarded perception as a precondition for the implementation of SAPs in terms of perception. Likewise, they said that its implementation leads to the perception of technology. Furthermore, Bopp [22] demonstrated that the implementation of SAPs will boost as farmers have a favorable perception of the adverse impacts of chemicals on health and the environment. Therefore, an existing study reported that the perceived significance of sustainable practices is an important factor influencing the implementation of SAPs [17].

In view of the aforesaid, this study makes the following hypotheses:

**H1.**  Socioeconomic status positively influences the adoption of SAPs.

**H2.**  Watching agricultural training programs positively influences the adoption of SAPs.

**H3.**  Newspaper and radio awareness campaigns positively influence the adoption of SAPs.

**H4.**  Participation in training extension courses positively influence the adoption of SAPs.

**H5.**  Sustainable agricultural perceptions positively influence the adoption of SAPs.

**H6.**  The feasibility of sustainable agricultural practices positively influences the adoption of SAPs.

**H7a.**  Attitudes influence the adoption of SAPs.

**H7b.**  Subjective norms influence the adoption of SAPs.

**H7c.**  Perceived behavior control influences the adoption of SAPs.

The last three have been split into three because they are components of behavioral intention.

### 2.3. Measurement of Key Variables

The primary dependent variable was adoption of SAPs. A household was considered an adopter if they at least adopted one of the SAPs. Accordingly, 1 represents an adopter and 0 otherwise. Such measurement of SAP adoption was also used by Mwalupaso [3].

On the other hand, socioeconomic and psychosocial factors were captured, and details are presented in Table 3. More importantly, control variables are also included in the interest of robust estimates, as exclusion is likely to lead to biased estimates according to Duguma and Han [35].

### 2.4. Analytical Framework and Empirical Strategy

The study used statistical packages for social sciences (SPSS) software to analyze the data. The logistic regression and SEM were applied to achieve the objectives of the study. 

The mathematical representation of the logistic regression is as follows [36]:

(1) $$Y=\{\begin{array}{cc} 1 & Y^{*}=\beta_{0}+\beta_{1}X_{1}+\beta_{2}X_{2}+\beta_{3}X_{3}+\beta_{4}X_{4}+\beta_{5}X_{5}+\beta_{6}X_{6}+\beta_{c}\;C_{i}+\epsilon \\ 0 & Otherwise \end{array},$$

where Y is the SAP adoption status, $\beta_{0}$ is the intercept, $\beta_{1}$ − $\beta_{6}$ are the coefficient associated with each independent variables $X_{1}$ − $X_{6}$ ($X_{1}$ = economy status, $X_{2}$ = watch agriculture training programs, $X_{3}$ = newspaper and radio awareness campaigns, $X_{4}$ = participation in training extension courses, $X_{5}$ = sustainable agricultural perceptions and $X_{6}$ = feasibility of sustainable agricultural perceptions), while $\beta_{c}$ is the coefficient of the control variables represent by $C_{i}$.

We tested the ability of the TPB to explain SAP adoption using the structural equation model. The path analysis was obtained using analysis of moment structures (AMOS) software (SPSS). However, as a reliability test, before analyzing the psychosocial factors, this study identified the measurement model criteria which deal with the three independent variables attitude, subjective norms and perceived behavioral control. The measurement model was assessed by confirmatory factor analysis (CFA). Related to validity and reliability, Bagozzi and Yi [37] proposed that the Cronbach’s alpha should be >0.70. Therefore, with ATT (Attitude) = 0.911, SN (Subjective Norms) = 0.902, INSAP (Adoption of sustainable agricultural practices) = 0.878 and PBC (Perceived Behavioral Control) = 0.806, all the measurement constructs are reliable and valid.

In addition, the average of variance extracted (AVE) estimates were greater than 0.6, and most of the square multiple correction values were greater than 0.5, as shown in Table 4 (above 0.36 is acceptable, and above 0.5 is ideal [38]). Therefore, the validity of the proposed constructs for the measurement appears to be satisfactory. Table 4 also shows that the measurement model has satisfactory discriminant validity because the square roots of AVE were higher than the values of its corresponding rows and columns, providing support for the discriminative validity [38]. For the goodness-of-fit (describing how well the statistical model fits a set of observations) index of the model, the test results were expressed as follows: χ2 = 122.074, df = 71, χ2/df = 1.71 < 5, CFI = 0.981, GFI = 0.951, AGFI = 0.927, NFI = 0.955, RFI = 0.942, IFI = 0.981, TLI = 0.975 and RMSEA = 0.047. Each variable had good reliability and validity, which allowed the analysis on the structural equation model.

## 3. Results

### 3.1. Adoption Intensity

Table 5 presents the classification of banana farmers based on the 23 SAPs provided in Table 2. Interestingly, 50.0% adopted 8–13 SAPs, while only 3.0% adopted a single practice. A good number of farmers also adopted more than 13 SAPs, but the adoption pattern suggests that there could be some barriers to the adoption of the different sets of practices.

### 3.2. Logistic Regression

Table 6 presents the logic model estimations, which include the independent variables and control variables. The findings show that all the hypotheses are positively and significantly correlated to SAP adoption. As shown, the coefficients of economy status, watching agriculture training programs, newspaper and radio awareness campaigns, participation in any training extension courses, sustainable agriculture perception and the feasibility of sustainable agricultural practices were found to have a positive influence on the adoption of SAPs. Likewise, age, education and credit access are the significant determinants of SAP adoption. Particularly, with an increase in age, the likelihood to adopt SAPs reduces, while auspicious access to credit and the attainment of better education increases the propensity to adopt SAPs.

The values of R^2^ of 0.724, with a fitness value of 40.620, shows the model’s capacity to reliably predict determinants of SAP adoption as explanatory variables, including an explanation of 72.4% percent of the variance in SAP adoption.

### 3.3. Structural Model Analysis

**Figure 3:**
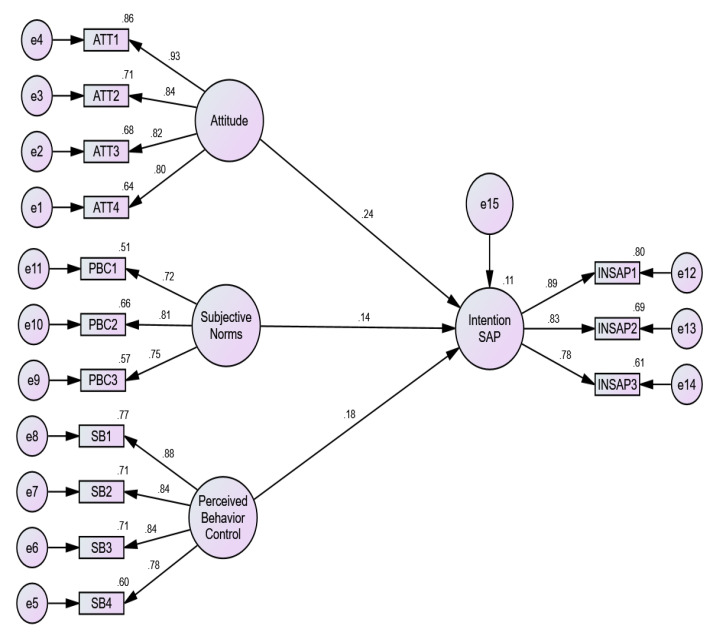
Structural model. Note: ATT = Attitude; SN = Subjective Norms; PBC = Perceived Behavior Control; INSAP, Intention to adopt Sustainable Agricultural Practices.

To measure the intention towards SAP adoption in banana production, structural equation modeling was applied. The reliability and validity of the model have already been presented. Using the Amos 24.0 software package, the goodness-of-fit indices of the model are as follows: χ2 = 146.724, df = 74, χ2/df = 1.982 < 5.0, RMSEA = 0.055 < 0.80, GFI = 0.941, AGFI = 0.917, CFI = 0.972, NFI = 0.946, RFI = 0.934 and TLI = 0.966. This indicates that the model has a satisfactory model goodness-of-fit. The results are provided in Table 7 and Figure 3.

The ATT had direct and significant impacts on INSAP, and their standardized path coefficients were 0.238 ** (*p* = 0.000). Thus, H7a was supported. Likewise, SN had direct and significant impacts on the INSAP, having standardized path coefficients in the order of 0.136 *** (*p* = 0.000). Hence, H7b was also supported. Finally, PBC had direct and significant effect on the INSAP, and its standardized path coefficient was 0.182 *** (*p* = 0.000), leading to the acceptance of H7c. Accordingly, consistent with the theory of planned behavior, ATT, SN and PBC regarding SAP adoption is key in promoting SAP adoption among banana farmers.

## 4. Discussion

The aim of this study was to identify the importance of socioeconomic and psychosocial factors on the adoption of sustainable agricultural practices among banana farmers. The impact is significant, and all the hypotheses were accepted. More to that, age, education and credit access are significant factors in promoting SAP adoption. Farmers’ age is often assumed to affect the implementation of SAPs [39]. Also, farmers’ schooling tends to have a positive impact on their choice to accept SAPs, although some studies found no impact on the acceptance, despite higher educational levels [40]. As concluded by Mwalupaso [3] and Guliyev [41], with advancement in age, farmers become too familiar with adopted practices and are thus unwilling to adopt new practices. On the other hand, education promotes and facilitates the adoption of new innovation. This was also found by Mwalupaso [3].

Regarding credit access, it is no surprise that increased access increases the propensity to adopt SAPs, because most small farmers are willing to adopt practices but are restricted by the cost of adoption. This was found to be true for farmers in Kazakhstan when assessing factors influencing adoption of certified seeds, which is also an SAP [42]. We will now look at the hypothesis of the study in detail.

### 4.1. Farmers’ Socioeconomic Status and Adoption of Sustainable Agricultural Practices

Economic status showed a substantial and positive effect on SAP adoption, consistent with previous studies [25]. The conclusion was that the economic status of farmers positively influences the adoption of SAPs. Mwalupaso [43] describes extreme severity (poverty line) as living on 2USD per day. This means that those below the poverty line are relatively poor, and thus any SAPs requiring farmers’ costly expenditure may not be adopted. In this sense, the cost of adoption stands as barrier for farmers who have a poor economic status. Therefore, to promote the adoption of expensive SAPs, farmers need assistance, because being economically better is linked with SAP adoption [4,14].

### 4.2. Watching Agriculture Training Programs and Adoption of Sustainable Agricultural Practices

More recently, researchers have indicated that agricultural training programs are directly associated with SAP adoption, because they provide effective and appropriate information to the farmers regarding methods to use in order to promote productivity in every aspect [44]. According to Ataei [45], agriculture training programs play a vital role in the development of production worldwide. Furthermore, these training programs provide a platform with a unique opportunity to not only learn about the farm industry, but also to learn about sustainable crop production [46]. It is important to note that watching training programs equips farmers with information, but may not demonstrate how to effectively execute the practice. This is most suitable for post-harvest and marketing practices. Therefore, farmers must be properly sensitized on what SAPs they can learn via observation, otherwise farmers may implement wrongly and end up with a perception that SAPs are not as effective as portrayed by SAPs adherents.

### 4.3. Newspaper Radio Awareness Campaign and Adoption of Sustainable Agricultural Practices

As newspapers and radio awareness provides basic information to farmers, the propensity to facilitate SAP adoption increases. Generally, sources of information play an important role in clarifying risks and uncertainties in the decision-making process. In this sense, it aids farmers in making good decisions regarding SAP adoption. In connection with implementing SAPs, various studies have demonstrated that TV and Radio have exposed farmers to mass media [47]. However, Thamaga-Chitja and Morojele [1] found that radio had no impact on the implementation of SAPs. Nevertheless, it must be noted that technological adoption is connected with extension policies and awareness, and these are efficient variables in the implementation of sustainable agricultural practices. Therefore, newspaper and radio campaigns must be a necessary information channel to provide guidance to farmers concerning any sustainable production on aspects covering crop and land management, fertilizer and pesticide practices, as well as harvest management [48].

### 4.4. Participation in Training Extension Courses and Adoption of Sustainable Agricultural Practices

According to Mirdamadi [49], participation in any training program related to sustainable agricultural practices is unavoidable for farmers to enhance their banana production. However, in Pakistan, the participation of farmers in training extension courses is less due to a lack of education among farmers and cultural barriers, especially for females [33]. Different modes of teaching are pivotal for successful training extension programs [50] because training programs can facilitate the effective execution of all the SAP categories in banana production. However, training may require financial incentives for farmers to participate, as exposed by Guliyev [41].

### 4.5. Sustainable Agriculture Perceptions and Adoption of Sustainable Agricultural Practices

The theory of planned behavior of social psychology is used to forecast and interpret particular behaviors [34]. This theory suggests that perceived difficulties in carrying out activities are a significant factor affecting adoption. Perception is a precondition for the implementation of SAPs, and it determines the decision. In support, Bopp [22] demonstrated that the implementation of SAPs is most likely when farmers have a favorable perception of the impacts of chemicals on health and the environment [17]. Similarly, our study found that the perceived impact of SAPs is an important factor influencing SAP adoption.

### 4.6. Feasibility of Sustainable Agriculture Practices and Adoption of Sustainable Agricultural Practices

The feasibility of SAPs is an essential aspect of SAP adoption. Rodriguez [51] discussed the beneficial impact of SAPs on production, but cited many barriers which make adoption not practicable. The feasibility has to do with a practice being achievable practically in the study area. Some SAPs may be very good but not practical due to many factors, such as lack of expertise, labor and policy support. Among factors affecting feasibility are labor availability and the cost of inputs [52]. While SAPs improve crop production, adoption is most likely influenced by whether the practices are practically possible. This implies that SAPs are location, crop and time bound.

### 4.7. Psychosocial Factors and Behavior towards Adoption of Sustainable Agricultural Practices

This study used TPB theory as the main theoretical framework to analyze farmers’ general attitudes toward SAPs [53]. The theory argues that a person’s intention is a good predictor of their actual behavior. According to this theory, a person’s attitude toward a behavior, subjective norms and their perceived behavioral control are the key antecedents of intention [34]. As earlier described, attitude is the extent to which a person has a favorable or unfavorable evaluation of a behavior. Subjective norms refer to an individual’s perceived social pressure to perform a certain behavior. It comprises beliefs about social expectations and the motivation to comply with those expectations [54]. Finally, perceived behavioral control is the extent to which an individual feels able to perform the behavior. This component addresses the issue of incomplete volitional control over individual actions, and must be carefully assessed when promoting the adoption of SAPs.

## 5. Conclusions

The general absence of the unconstrained selection of SAPs among smallholder farmers has been a significant concern for researchers and policymakers alike. A few research enquiries have endeavored to comprehend the elements obstructing or encouraging the take-up of SAPs. Research has transcendently centered around financial drivers, with little accentuation of the psychosocial and socioeconomic factors influencing farmers’ innovation inclinations. The examination in this study comprised an integrative methodology, breaking down how a blend of socioeconomic and psychosocial variables influences SAP adoption by smallholder farmers dealing with an important fruit (banana). Therefore, the purpose of this study was to investigate the impact of socioeconomic and psychosocial factors on SAPs using logistic regression and structural equation model. The study was underpinned by the theory of planed behavior, which places strong emphasis on three psychosocial factors that facilitate adoption - attitude, subjective norms and perceived behavioral control. To the best of our knowledge, this is an understudied area, as no study has ever assessed the impact of socioeconomic and psychosocial factors on SAP espousal.

The results reveal that age, education and credit access are significant in promoting SAP adoption. We also found that all the socioeconomic and psychosocial factors assessed are significantly correlated with adoption, but caution must be exercised on which particular SAPs can be promoted using the studied extension methods. Therefore, we recommend that interdisciplinary and all-encompassing methodologies should frame some portion of compelling procedures for advancing SAPs among smallholder banana farmers, concentrating on both socioeconomic and psychosocial factors. Methodologies to improve adoption rates could incorporate the arrangement of money related motivators to assist with initializing speculation and opportunity costs. This will accentuate custom-made expansion benefits which give applicable data on SAPs and therefore help dissipate normal misguided judgments and abbreviate the learning circle.

Finally, this study has some limitations that also provide opportunities for future studies. First, the study obtained a sample representation of banana farmers from the Sindh province of Pakistan only. The limited number of participants might constrain our generalization of results. Second, this study explores the influence of socioeconomic and psychosocial factors on the adoption of SAPs with a focus on the existing barriers to SAP adoption. Nevertheless, in view of the dearth of empirical evidence on the subject, the study provides vital information that could inform policy. To corroborate our finding, future studies should make use of panel data on barriers, risk, policy variables, cost-share payment and other important socioeconomic factors to establish more robust conclusions on the causality of these variables in determining the adoption or adoption intensity of SAPs in Pakistan.

## Figures and Tables

**Table 1:** Latest studies on farmer’s adoption of sustainable agricultural practices.

Authors and Year	Country	Analysis Technique	Findings
Mutyasira, Hoag [17]	South Africa	Data were collected from 359 smallholder farmers using questionnaires.	Traditional sustainable agricultural practices such as intercropping, mulching and crop rotation were more likely to be adopted by farmers.
Bopp, Engler [22]	Chile	A count model was estimated.	Extrinsic motivation factors have a positive impact on the adoption of sustainable agricultural practices among farmers”.
Adnan, Nordin [23]	Malaysia	Analyzed three phases of sustainable agricultural practices among Malaysian Paddy farmers.	SAPs have a positive impact on agricultural outcomes
Mutyasira, Hoag [24]	Ethiopia	Ordered Probit model and Partial Least Squares Structural Equation Modeling (PLS-SEM) to model farmers’ adoption decisions.	Farmers’ intentions and personal norms significantly influence the number of SAPs adopted at farm-level.
Nkomoki, Bavorová [25]	Zambia	The sample consisted of 400 households, 200 with customary and 200 with statutory land tenure.	Land ownership influences the adoption of SAPs.
Tey [26]	Malaysia	Utilized informational sources and other key identified factors affecting Malaysian vegetable producers’ choices.	Information sources are complementary and influenced by heterogeneity in access to credit, social network and farm settings.

**Table 2:** Uses of measures during banana production.

Category	Adoption Rate (%)
**Land Management**	
Limiting tilling and hoeing	52.3
No pasturing cattle in production areas	50.7
**Crop Management**	
Seedlings controlled diseases by chemicals before growing	53.3
Use of improved banana varieties	50.7
Irrigation	52.3
Rotation of crops	52.0
No-chemical weed control	53.0
Application of indigenous knowledge	52.0
Crop intercrop (legumes or annual crops)	52.7
Mulches	50.0
Participating in farmer groups	54.7
**Pesticide Practices**	
Biological pest and diseases control	54.7
Proper use of pesticides	49.3
**Fertilizer Practices**	
Application of inorganic fertilizers	45.0
Application of organic fertilizers (green and animal manure)	54.0
Conducting soil test before applying fertilizers	49.7
**Harvest Management**	
No fruits touch the land after harvesting	52.0
Using fresh equipment to harvest fruits	52.0
Fruits harvested at maturity	53.0
**Post-Harvest and Marketing**	
Fruits preserved in fresh materials	43.7
Products sold to enterprises through contractual agreement	54.0
Products registered with label showing ‘met quality standards’	52.0
Regular products quality checks by relevant authorities	49.7

**Table 3:** Measurement of socioeconomic and psychosocial factors.

Variable Category	Description and Measurement
**Socioeconomic Factors**	
Economy Status	Above poverty Line = 1, Otherwise = 0
Watch Agricultural Training Program	Yes = 1, Otherwise = 0
Newspaper and radio awareness campaigns regarding agriculture training program	Yes = 1, Otherwise=0
Participated in any training extension course	Yes = 1, Otherwise = 0
Sustainable agricultural perception	Yes = 1, Otherwise = 0
Feasibility of sustainable agricultural practices	Yes = 1, Otherwise = 0
**Psychosocial Factors**	
Attitude towards SAP adoption	1 = negative, 2 = neutral, 3 = positive
Subjective norms	Assessed by the perceived social pressure influencing individual behavior, and identifying the most influential person on their adoption, regarding four referents: family, peer groups, neighbors, and the government (1 = strongly disagree, 2 = disagree, 3 = neutral, 4 = agree and 5 = strongly agree).
Perceived behavioral control	Assessed using the scale of self-confidence to adjust current farming practices in the next 3years (1 = strongly disagree, 2 = disagree, 3 = neutral, 4 = agree and 5 = strongly agree)
**Control Variables**	
Age	The age of the household head in years
Labor Household	The number of people in a household who are part of the labor force
Education	The number of years of schooling of the household head
Experience	The number of years of farming experience
Ethnic Group	The tribe of the household (1 = Urdu and 0 otherwise)
Farm Size	The size of the farm used for banana cultivation
Labor Assess	The availability of hired labor (1 = accessible and 0 otherwise)
Machine Access	Accessibility to use machines in banana production (1 = have access, 0 otherwise)
Fertilizer use	Use of chemical fertilizer in banana production (1 = user, 0 otherwise)
Pesticide use	Use of pesticides in banana production (1 = user, 0 otherwise)
Credit Access	Accessibility to credit (1 = have access, 0 otherwise)

**Table 4:** Reliability and Validity Test.

Key Variables	CR	AVE	MSV	MaxR(H)	ATT	SN	INSAP	PBC
ATT	0.911	0.721	0.072	0.925	0.849			
SN	0.902	0.696	0.072	0.906	0.113 ^†^	0.835		
INSAP	0.878	0.706	0.072	0.889	0.269 ***	0.233 ***	0.840	
PBC	0.806	0.581	0.072	0.817	0.142*	0.269 ***	0.215 **	0.762

Notes: CR = Composite reliability; AVE = Average variance extracted; MSV = Maximum shared variance; MaxR(H) = maximum reliability; ATT = Attitude; SN = Subjective Norms; PBC = Perceived Behavior Control; INSAP, Intention to adopt Sustainable Agricultural Practices. Significance of Correlations ^†^ *p* < 0.100, * *p* < 0.050, ** *p* < 0.010 and *** *p* < 0.001.

**Table 5:** Classification of banana farmers based on the number of SAPs adopted.

SAP adoption Intensity	Description	Frequency	Percent
Low	Farmers who adopted 1 practice	9	3.0
Fairly Low	Farmers who adopted 2–7 practices	56	18.7
Fairly High	Farmers who adopted 8–13 practices	150	50.0
High	Farmers who adopted above 13 practices	85	28.3

**Table 6:** Results of LR analysis.

Explanatory Variables		Beta	t	*p*	Collinearity Statistics	Decision
X1	Economy status	0.119 ***	3.778	0.000	1.969	Accepted
X2	Watch agriculture training program	0.132 ***	4.340	0.000	1.663	Accepted
X3	Newspaper awareness and radio campaign regarding agriculture training program	0.145 ***	4.859	0.000	1.745	Accepted
X4	Participated in any training extension course and SAPs	0.124 ***	4.012	0.000	1.870	Accepted
X5	Sustainable agricultural perception	0.198 ***	7.921	0.000	1.310	Accepted
X6	Feasibility of sustainable agricultural practices	0.101 ***	3.093	0.002	2.060	Accepted
C1	Age	−0.036 *	1.409	0.100	1.056	Accepted
C2	Labor Household	−0.036	−1.281	0.201	1.170	Rejected
C3	Education	0.047 **	−1.933	0.004	1.297	Accepted
C4	Experience	10.033	−1.504	0.134	1.631	Rejected
C5	Ethnic Group	−0.024	−0.903	0.367	1.145	Rejected
C6	Farm Size	0.027	1.125	0.261	1.793	Rejected
C7	Labor Assess	0.039	0.933	0.352	1.211	Rejected
C8	Machine Access	−0.052	−1.151	0.251	1.272	Rejected
C9	Fertilizer use	0.038	0.871	0.385	1.332	Rejected
C10	Pesticide use	−0.031	−0.747	0.456	1.242	Rejected
C11	Credit Access	0.008 ***	−0.192	0.000	1.085	Accepted

Notes: R^2^ = 0.724; Adjusted R^2^ = 0.695, F = 114.728; *** *p* < 0.01, ** *p* < 0.05, * *p* < 0.1.

**Table 7:** SEM estimation.

Outcome Variable		TPB Aspect	Estimate	S.E.	C.R.	*p*
Intention SAP	<---	Attitude	0.238	0.089	3.990	0.000 ***
Intention SAP	<---	Subjective Norms	0.136	0.089	2.167	0.030 **
Intention SAP	<---	Perceived Behavior Control	0.182	0.090	3.041	0.002 ***

Notes: S.E is the standard errors while C.R is composite reliability. <--- means that there is a relationship with the outcome variable. *** *p* < 0.01, and ** *p* < 0.05.
